# Editorial: Advances and challenges in pediatric immune disorders: from pathogenesis to personalized therapy

**DOI:** 10.3389/fped.2026.1944386

**Published:** 2026-08-13

**Authors:** Yan Pan, Fuyong Jiao, Hong Wang, Weihua Zhang, Bilal Haider Shamsi

**Affiliations:** 1 Department of Pediatrics, The First Affiliated Hospital of Yangtze University, Jingzhou, China; 2 Shaanxi Provincial People’s Hospital, Xi’an, China; 3 Shengjing Hospital of China Medical University, Shenyang, China; 4 Xianyang Caihong Hospital, Xianyang, China; 5 Rawalpindi Medical University, Rawalpindi, Pakistan

**Keywords:** autoimmune disease, biomarkers, inborn errors of immunity, kawasaki disease, neuroinflammation, pediatric immune disorders, personalized therapy

Childhood is a uniquely vulnerable period during which the immune system is still being calibrated: too little immunity invites catastrophic infection, whereas too much or misdirected immunity drives vasculitis, autoimmunity, autoinflammation and neuroinflammatory injury. Pediatric immune disorders therefore sit at the crossroads of immunology, infectious diseases, genetics and precision medicine, and their management increasingly demands that clinicians move beyond syndromic labels toward mechanism-informed, individualized care. This Research Topic, *Advances and Challenges in Pediatric Immune Disorders: From Pathogenesis to Personalized Therapy*, brings together 15 contributions—seven original research articles, six case reports, one review and one perspective—that collectively map this transition. Rather than providing a simple catalog, the articles in this Research Topic converge on several unifying questions: how can we recognize high-risk children earlier, how do host immunity, microbes and metabolism interact to shape disease, and how can treatment be tailored to the individual child while remaining feasible in real-world settings?

Kawasaki disease (KD), the leading cause of acquired heart disease in children in developed countries, emerges as the thematic center of gravity of this Special Issue, examined from different angles. In their perspective article, Yi et al. argued that KD should be reframed not as a self-limited febrile illness but as a chronic disease model, in which an acute “immune storm” imprints lifelong vascular risk and calls for structured, decades-long cardiovascular surveillance. This view aligns with growing evidence that coronary and systemic endothelial sequelae may persist well beyond the acute phase.

Where does the acute storm originate? Zhao et al. provided the first systematic multi-omics characterization of the gut microbiota and metabolome of children with KD in Northwest China. The authors found depleted microbial diversity and an increase in potentially pathogenic species such as *Enterococcus avium*, *Streptococcus peroris* and *Clostridioides difficile*. There was also a loss of butyrate-producing commensals, including *Faecalibacterium prausnitzii*, accompanied by reduced fecal butyrate and an increase in pro-inflammatory plasma metabolites. These findings position the gut–immune–vascular axis as both a source of candidate biomarkers and a potential therapeutic target.

Risk stratification, however, must also work where resources are limited. Li et al. built predictive models and nomograms for incomplete KD and coronary artery lesions using only routine laboratory indicators, identifying hypoalbuminemia as the strongest independent predictor of coronary injury, together with hyponatremia and elevated lactate dehydrogenase (AUC 0.790). Such inexpensive tools are particularly valuable for primary care. The case reported by Boyarchuk et al.—a child with a congenital coronary artery anomaly that presented a diagnostic dilemma between KD and multisystem inflammatory syndrome in children—reminds us that pre-existing anatomy can profoundly affect how immune-mediated vasculitis declares itself. Complementing this vascular theme, Chengjun Zhang et al. showed, using cardiac magnetic resonance, that elevated homocysteine is independently associated with reduced ejection fraction in children with myocarditis, especially in boys and those overweight, adding a measurable metabolic dimension to cardiac risk assessment in inflammatory heart disease.

A recurring message of this Research Topic is that infection and immune dysregulation are not opposing diagnoses but rather frequent accomplices. He et al. described an 11-year-old boy with IgA vasculitis who developed varicella-zoster virus–induced hemophagocytic lymphohistiocytosis complicated by disseminated intravascular coagulation and acute liver failure. Treatment required a carefully staged strategy—high-dose acyclovir, intravenous immunoglobulin, plasma exchange, and reduced-dose etoposide—illustrating that the therapeutic goal in infection-triggered hyperinflammation is not maximal immunosuppression but rather calibrated immunomodulation. The authors’ observation that lymphocytopenia persisted for 2 months after discharge further suggests lymphocyte recovery as a practical biomarker of immune reconstitution. The flip side of the same coin was presented by Liang et al.: a 16-year-old with Crohn's disease who developed viral encephalitis during adalimumab therapy, identified through metagenomic next-generation sequencing, a warning that biologic-induced immunosuppression can unmask opportunistic neurotropic infections. Finally, Yang et al. analyzed 56 children with invasive pneumococcal disease and identified procalcitonin and the neutrophil-to-lymphocyte ratio as independent predictors of purulent meningitis (combined AUC 0.885); notably, more than half of the children who developed meningitis had underlying immunocompromise, reinforcing that host immune status conditions bacterial invasion of the central nervous system.

Neuroimmunology is one of the fastest-moving areas of pediatric immunology, and three contributions chart its clinical frontier. In a retrospective cohort of 63 children with myelin oligodendrocyte glycoprotein antibody–associated disease (MOGAD), Pei et al. demonstrated that persistently positive serum MOG-IgG is associated with relapse and poorer long-term disability, whereas monophasic disease is associated with earlier seronegativity; high titers at onset correlated with greater cerebrospinal fluid pleocytosis and longer glucocorticoid exposure. These data support incorporating serial antibody monitoring into treatment-duration decisions—an actionable step toward personalized neuroimmunology. Linlin Zhang et al. characterized autoimmune encephalitis associated with antibodies against intracellular antigens in children, a subgroup that is easily missed by cell-based assays designed for surface antigens and in which seizures dominate the clinical picture; their study clarifies the immunological basis, antibody spectrum, and therapeutic approaches for this under-recognized entity.

Precision diagnosis increasingly begins in the genome. Dai et al. reported on a child with hyper-IgE syndrome caused by a STAT3 c.1915C > T variant, expanding the mutational landscape of this inborn error of immunity (IEI) and highlighting the value of genetic confirmation in atypical presentations. Shulan Zhang et al. discovered hidden B-cell dysregulation in MIRAGE syndrome through the transcriptomic profiling of identical twins, revealing the depletion of memory and naïve B cells, the expansion of CD8^+^ T cells and M2 macrophages, and the enrichment of PI3K-Akt and B-cell receptor signaling pathways—evidence that a syndrome defined by growth restriction and myelodysplasia also carries a clinically relevant humoral defect with infection-prevention implications. Rare disease in underrepresented populations was exemplified by Luo et al., who described a young Chinese man with cystic fibrosis complicated by *Mycobacterium abscessus* infection. This case illustrates the distinct CFTR mutation spectrum in Chinese patients, the value of metagenomic sequencing in unraveling chronic infection, and—soberingly—the treatment gap created when CFTR modulators remain financially inaccessible.

Two contributions addressed the destination of this journey: choosing and delivering the right treatment for the right child. Rodríguez-Lozano et al. prospectively compared the ACR-1997, SLICC-2012, and ACR/EULAR-2019 classification criteria in Mexican children with suspected childhood-onset systemic lupus erythematosus, adding a rare economic dimension to a familiar methodological debate. SLICC-2012 was the most sensitive (98%), whereas ACR-1997 was the most cost-effective; the authors pragmatically recommend matching the instrument to the setting—sensitive criteria to aid referral in non-specialized centers and parsimonious criteria where expertise is concentrated. In their review, Yin et al. surveyed the expanding therapeutic armamentarium for pediatric autoimmune disease, ranging from conventional biologics targeting TNF, IL-6 and BAFF to chimeric antigen receptor cell therapies. The review clarifies that the prospect of deep, drug-free remission is mitigated by pediatric-specific uncertainties, such as age-dependent pharmacology, infection risk, long-term safety, and profound global inequities in access.

Taken together, the 15 articles in this Research Topic present a coherent trajectory for pediatric immunology. First, biomarker-guided risk stratification—whether a nomogram built on albumin and sodium, a serial antibody titer, or a metabolomic signature—is steadily replacing one-size-fits-all thresholds. Second, the pathogenesis of pediatric immune disease is increasingly understood as an ecosystem phenomenon, in which gut microbes, metabolites, host genetics, and environmental triggers interact; single-agent explanations are giving way to network models. Third, every advance in immunosuppression creates a parallel obligation to anticipate infection, and staged, risk-adapted immunomodulation is emerging as the dominant therapeutic philosophy. Finally, precision medicine will fail to deliver on its promise if its tools remain inaccessible: several contributions, from cystic fibrosis care in China to lupus classification in Mexico, underscore that feasibility and cost-effectiveness are scientific questions in their own right.

We thank all the authors and reviewers who contributed to this Research Topic. We hope that this collection stimulates multicenter, prospective, and mechanistically anchored studies—and, above all, better outcomes for the children whose immune systems are still learning what to fight and what to spare.

